# New Advances in Neuropsychiatric Disorders of Childhood and Adolescence

**DOI:** 10.3390/brainsci12030399

**Published:** 2022-03-16

**Authors:** Michele Roccella, Luigi Vetri

**Affiliations:** 1 Department of Psychology, Educational Science and Human Movement, University of Palermo, 90128 Palermo, Italy; michele.roccella@unipa.it; 2 Oasi Research Institute—IRCCS, Via Conte Ruggero 73, 94018 Troina, Italy

Neurological and psychiatric disorders during developmental ages affect an increasing share of the pediatric population, both due to the increased understanding and attention paid to these issues and due to increased risk factors. There exist different pathologies, from infantile cerebral palsy to autism, from language and learning disorders to psychomotor and mental disability, from epilepsy to mood disorders and psychosis, from phobias and anxiety disorders to behavioral, sleep and food disorders and sphincter dysfunction, and from attention deficit hyperactivity disorder to neuromuscular pathologies and movement disorders.

At every stage of development, it is important to make an early and correct diagnosis that evaluates not only the instrumental elements, but also and above all the psychomotor, affective, cognitive and relational development of the child. This diagnosis allows us to set up targeted and early therapeutic and rehabilitative interventions, which take into account the specific developmental stage and also any associated disorders, supporting the family and extra-family context and the school context in which the child or the adolescent lives. Early intervention is a fundamental element in order to make the most of windows of therapeutic opportunities, provided by the brain plasticity of the developmental age.

Therefore, in order to maximize the therapeutic results, it is essential to provide a more global approach to the child’s care that takes into account the neuromotor, neurocognitive and psychological profile, including the overall response and time taken to respond to therapeutic and rehabilitative treatment.

The research articles and literature reviews of the Special Issue “*Neurological Diseases in Children Series II*” are important evidence of the complexity of neuropsychiatric disorders in childhood and adolescence.

Caliendo et al., in their multicenter study, analyzed the effects of six months of neuro-psychomotor treatment in children with autism spectrum disorder and its impact on different aspects of life. After six months of neuro-psychomotor treatment, children with ASD were evaluated by the ASD behavior inventory (ASDBI), and they showed a significant reduction in the domain problems of excitability, aggression, behaviors in social scenarios, expressiveness, sense/perceptual contact modes, ritualism/resistance to change, pragmatic/social problems, specific fears, learning and memory, while there were no differences in the domains of semantic/pragmatic problems and language [1].

Operto et al. in their study assessed the cognitive and emotional–behavioral functioning and parental stress of children with neurodevelopmental disorders (autism spectrum disorder level 1/high-functioning, specific learning disorders and attention deficit/hyperactivity disorder). The authors found that in the three neurodevelopmental disorders considered there is a peculiar neuropsychological cognitive profile. In particular, the global intelligence autism spectrum disorder level 1/high-functioning group scored higher than ADHD and learning disorders groups, the subjects with learning disorders have more impaired working memory and processing speed skills. The autism spectrum disorder level 1/high-functioning group displayed good performance in logical reasoning and weakness in processing speed; ADHD subjects had more difficulties in verbal comprehension, working memory and processing speed abilities; emotional–behavioral problems were present in all groups compared to controls, plus all the parents of children with neurodevelopmental disorders showed higher levels of stress than parents of typically developing children [2]. 

In the current Special Issue, particular attention was paid to restricted and repetitive behaviors and interests that are among the core symptoms of autism spectrum disorder. Lanzarini et al. in their observational and retrospective study investigated the frequency, variability, and typologies of phonic and motor stereotypies in children with ASD and their association with clinical neurological variables. Children with ASD and cognitive impairment showed a higher number and variability of motor stereotypies involving the head, trunk and shoulders. Noncommunicative stereotypies were more frequent in nonverbal patients, while echolalic stereotypies were more prevalent in verbal patients, guttural stereotypies were significantly more frequent in children with a higher variability of motor stereotypies and with self-directed motor stereotypies, while visual/gaze stereotypies were more frequent among children with a smaller presence of phonic stereotypies [3]. 

In the same area of investigation, Pruccoli et al. reviewed the existing literature on a particular form of phonic restrictive and repetitive behaviors of autism spectrum disorder: echolalia and its role in the development of children with ASD. A total of 76 articles were included in the review, and several relevant studies documented a developmental role of echolalia in ASD children with the aim of facilitating the acquisition of verbal skills. Treating echolalia as a troubling symptom could impair language development. According to this evidence, authors suggest considering echolalia not as restrictive and repetitive behaviors but rather as an atypical social communication feature of children with ASD [4]. 

Fetta et al., in their retrospective–prospective observational study, evaluated the relationship between repetitive behaviors and sensory profile. Authors administered to the caregivers of children with ASD the Repetitive Behaviour Scale-Revised and the Short Sensory Profile (SSP) and found the strongest correlation between visual/auditory sensitivity and stereotyped and sameness behavior, and between under-responsive/seeks stereotypical and repeated behaviors. These results can help to improve the effectiveness of treatment strategies to mitigate the impact that these sensory alterations could produce in behavioral manifestations of children with ASD [5]. 

A plethora of studies have demonstrated that primary headaches are increasingly common conditions in children and adolescents. Cesaroni et al., in their review, analyzed the literature evidence about the key features of therapeutic options, during the developmental age, of Short-lasting Unilateral Neuralgiform headache attacks with Conjunctival injection and Tearing (SUNCT) and Short-lasting Unilateral Neuralgiform headache attacks with cranial Autonomic symptoms (SUNA), rare primary headache syndromes belonging to the group of Trigeminal Autonomic Cephalalgias. Thanks to a review of 60 articles, the authors described the pathophysiology of SUNCT and SUNA and analyzed the therapeutic options available to date, especially underlining less-invasive and risk-free therapies as possible treatment options for pediatric patients [6]. 

In summary, the Special Issue “*Neurological Diseases in Children Series II*” has provided us with interesting insights about neurodevelopmental disorders, conditions with numerous open questions regarding their etiology and treatment fields in which there is still plenty of room for further research.
